# A Review of Preventive and Social Medicine: By Chandrakant Lahariya, India

**Published:** 2009-01

**Authors:** Binod Kumar Patro

**Affiliations:** Assistant Professor of Community Medicine, PGIMER School of Public Health, Chandiagarh, India. E-mail: patrobinod@yahoo.co.in

This is a welcome addition to the list of books available in the discipline of Preventive Medicine/Community Medicine/Public Health. The book covers both conventional and advanced public health issues with equal vigor and enthusiasm. The author has arbitrarily categorized all the reviews into ten units. Fundamental questions asked by new entrants to the aforementioned disciplines are given in unit 1 of the book, aptly named as, “The Basics.” Important issues of public health/preventive and social medicine such as “Essential Medicines”, “International Health Regulations”, as well as “Poverty and Health” which are usually not covered in conventional text books, find a place in this book. Newer topics like “Genetically Modified Foods and Health” and “The Air Ticket Levy” are placed appropriately in the book. The flagship program of the Government of India, the “National Rural Health Mission” is covered in detail. The unit on “Social Medicine” covers a mixed group of topics staring from “Qualitative Research Methods” to management issues like “Medical Audit and Social Marketing.” The chapter on “District Level Household Survey 2002–04” is arbitrarily placed in the “Social Medicine” unit, while “National Family Health Survey-III” is placed within the “Health” unit. The chapter on “Health Economics: The basics” covers little less than the basics, which need elaboration. Certain chapters like “Health Economics,” “Small Pox,” “Health Sector Reforms,” “Design Effect,” “Social Audit and Untied Fund,” and “Indian Public Health Standards” do not have any bibliography which might put the reader at some disadvantage.

Overall, the book has a good layout and makes good reading. The book has a good index spreading over 12 pages. Considering the nature of the contents, the book requires periodic revision. The size of the book stands between that of a pocket book and a standard textbook. Priced modestly, the book can be an additional resource material for students of community medicine, public health, and the social sciences.

